# Current News

**Published:** 1904-04

**Authors:** 


### Current News.

### NEW JERSEY STATE BOARD OF REGISTRATION AND EXAMINATION IN DENTISTRY.

The New Jersey State Board of Registration and Examination in Dentistry will hold its next examination in the theoretical branches in the Assembly-room of the State-House at Trenton, N. J., on the 5th, 6th, and 7th of July, sessions beginning promptly at nine A.M.

The practical work done in Newark. All applications must be in the hands of the Secretary ten days prior to the examination.

For information regarding the examination, apply to the Secretary, 29 Fulton Street, Newark, N. J.

Charles A. Meeker,
*Secretary and Treasurer.*

### KENTUCKY STATE DENTAL ASSOCIATION.

The coming annual meeting of the Kentucky State Dental Association promises a dental convention of unusual interest; to be held in Louisville May 17, 18, and 19, 1904.

Members of the profession are extended a hearty welcome.

W. M. Randall,
*Secretary.*

Masonic Building, Louisville, Ky.

### MONTANA STATE DENTAL SOCIETY.

The first annual meeting of the Montana State Dental Society was held in Helena, Mont., February 22 and 23, 1904, and the following officers were elected for the coming year: President, Dr. W.

H. Barth, Great Falls; First Vice-President, Dr. J. D. Sutphen, Helena; Second Vice-President, Dr. Joseph Oettinger, Missoula; Secretary, Dr. George E. Longeway, Great Falls; Treasurer, Dr. W. M. Billings, Helena.

The second annual meeting will be held in Butte, Mont., February 20 and 21, 1905.

George E. Longeway,
*Secretary.*

### CENTRAL DENTAL ASSOCIATION OF NORTHERN NEW JERSEY.

At the annual meeting of the Central Dental Association of Northern New Jersey, held at Newark, February 15, 1904, the folowing officers were elected:

President, C. S. Stockton, Newark; Vice-President, T. Starr Dunning, Paterson; Secretary, H. Parker Marshall, Newark; Treasurer, C. A. Meeker, Newark.

*Executive Committee.*—R. S. Sanger, East Orange, Chairman; W. Moore Gould, Newark, Secretary; N. M. Chitterling, Bloomfield; Frank L. Manning, Red Bank; Frederick W. Stevens, Newark.

Frederick W. Stevens,
*Secretary.*

### ILLINOIS STATE DENTAL SOCIETY.

The fortieth annual meeting of the Illinois State Dental Society will be held at Peoria, Tuesday, Wednesday, and Thursday, May 10, 11, and 12, 1904. A splendid programme, including attractive and unusually interesting features, is under course of preparation. The usual fare of one and one-third—certificate plan—will be obtained on all roads in the State and from St. Louis. Remember the date. All reputable practitioners cordially invited.

Hart J. Goslee,
*Secretary.*

### FIRST DISTRICT DENTAL SOCIETY OF NEW YORK.

THE following officers have been elected for 1904:

President, Dr. Henry D. Hatch; Vice-President, Dr. Arthur L. Swift; Secretary, Dr. Benj. C. Nash; Treasurer, Dr. James W. Taylor; Librarian, Dr. F. L. Stanton.

*Executive Committee.*—Dr. Wm. C. Deane, Dr. F. L. Fossume, Dr. Wm. Carr, Chairman.

*Clinic Committee.*—Dr. W. D. Tracy, Dr. Ralph B. Reitz, Dr. S. L. Goldsmith, Chairman.

B. C. NASH,
*Secretary.*

